# On the Cause of Death in Membranous Inflammations

**Published:** 1831

**Authors:** 


### On the Cause of Death in Membranous Inflammations. By M. Broussais.

The following are the conclusions to which the eccentric, but talented, Professor of Val de Grace has come on the above topic.

1. The membranous phlegmasiæ, more especially those of the abdomen, particularly gastritis and enteritis, frequently determine, even at their commencement, congestion in the brain and spinal marrow, sufficient to destroy life, if proper means be not promptly employed. This congestion often, as, for example, in infants, becomes the principal disease.

2. During the course of these inflammations, there exists *always* a sympathetic irritation of the brain and spinal marrow, which more or less accelerates the circulation and respiration, disturbs the sensorial functions, perverts the sensations, renders the voluntary muscles unfit for their offices, and, in a word, occasions all those nervous symptoms which we see in this class of diseases, even when they are most mild.

3. When these phlegmasiæ have profoundly altered the mucous membranes, and menace a fatal termination, very severe nervous symptoms are developed, such as delirium and convulsions, when the membranes of the brain are the chief seat of the sympathetic irritation—or coma, when the central parts of the cerebral mass are affected, and exhalation takes place into the cavities of the brain.

4. It is this irritation which always leaves traces of inflammatory action, that terminates life, *by functional disturbance*, whenever death occurs before encephalic effusion, hæmorrhage, or organic change in the great vital organs. It may be asserted that peritonitis, even where there is perforation of the gut, does not, in itself, occasion death, but only through the constitutional disturbance of the brain and nervous system, as before stated.

5. Finally, M. Broussais concludes that those alterations of structure which we see in acute diseases, hitherto called idiopathic fevers, are the effects of inflammation—and if, in certain parts of the said membrane, there be appearances of paleness, or even atrophy, these he attributes to the afflux of blood to various other parts of the body in articulo mortis, or during the last day or two of life.—*Annales de la Médécine.*

---

